# Classification and Treatment of Pediatric Gliomas in the Molecular Era

**DOI:** 10.3390/children8090739

**Published:** 2021-08-27

**Authors:** Peter Hauser

**Affiliations:** Velkey László Child’s Health Center, Borsod-Abaúj-Zemplén County Central Hospital and University Teaching Hospital, Szentpéteri kapu 72–76, 3526 Miskolc, Hungary; hauserpeti@yahoo.com

**Keywords:** child, classification, central nervous system (CNS) tumor, diffuse intrinsic pontine glioma (DIPG), glioma, glioblastoma, high grade glioma, low grade glioma, mitogen-activated protein kinase (MAPK), targeted therapy

## Abstract

The overall survival of pediatric gliomas varies over a wide spectrum depending on the tumor grade. Low-grade gliomas have an excellent long-term survival, with a possible burden of surgery, irradiation, and chemotherapy; in contrast, high-grade gliomas generally have a short-term, devastating lethal outcome. Recent advances in understanding their molecular background will transform the classification and therapeutic approaches of pediatric gliomas. Molecularly targeted treatments may acquire a leading role in the primary treatment of low-grade gliomas and may provide alternative therapeutic strategies for high-grade glioma cases in the attempt to avoid the highly unsuccessful conventional therapeutic approaches. This review aims to overview this progress.

## 1. Introduction

Malignant CNS (central nervous system) tumors are the second most common cancers of childhood, constituting 21% of cases. Gliomas are the most common type of CNS tumors, accounting for 35% of CNS tumors diagnosed in children between birth and 19 years of age [1]. Pediatric gliomas consist of WHO (World Health Organization) histological grade 1–2, low-grade gliomas (pLGG) (e.g., pilocytic astrocytomas, diffuse gliomas), accounting for 25% to 30% of all childhood CNS tumors, and WHO histological grade 3–4, pediatric high-grade gliomas (pHGG) (e.g., anaplastic astrocytoma, GBM (glioblastoma), DIPG (diffuse intrinsic pontine glioma)), accounting for one-third of pediatric gliomas [2]. Conventional therapies including neurosurgical intervention, radiotherapy, and chemotherapy often provide poor post-treatment quality of life, and survival of glioma patients is still devastating in several cases. In general, pLGGs have more than 90% overall survival rate; on the contrary, pHGGs have less than 10% long-term survival rate, despite the aggressive treatment regimens [3,4]. Recently, clarification of the molecular background of different gliomas has opened a new era in terms of their classification and treatment. In this review, this progress will be overviewed, offering a new hope for children with gliomas.

## 2. Epidemiology and Histological Classification

The latest WHO classification of CNS tumors was published in 2016, introducing some molecular parameters [5]. This classification considered growth pattern, tumor behavior, and shared genetic driver mutations to group different entities, in contrast to the previous WHO 2007 classification that was only focused on cellular origin [5]. The WHO 2016 classification only emphasized the significance of H3K27M mutation as a genetic driver mutation in pediatric gliomas, as IDH (isocitrate dehydrogenase) mutation or 1p/19q codeletion is extremely rare in childhood [6].

pLGGs or glioneuronal tumors (WHO grade 1 or 2) are highly heterogeneous entities. The most common single entity is pilocytic astrocytoma (0.91/100.000 patients age 0 to 19 years), followed by ganglioglioma, dysembryoplastic neuroepithelial tumor (DNET), and Grade 2 diffuse gliomas. High-grade gliomas of childhood are a histologically less heterogenic group of tumors with lower incidence compared to pLGGs (age-adjusted incidence, 0–19 years of age, is 0.26/100.000 without DIPGs) [7]. pHGGs consist of Grade 3 tumors like anaplastic astrocytomas (0.1/100.000 patients age 0 to 19 years) and anaplastic gangliogliomas, and Grade 4 tumors include GBM (0.18/100.000 patients age 0 to 19 years), DIPG (80% of brain stem tumors, which accounts for 15% of all CNS tumors), and gliomatosis cerebri, which is a highly infiltrative, special manifestation of HGG affecting multiple brain regions, not regarded as a separate subgroup any more [7,8].

Recent advances in next-generation sequencing and array-based genomic platforms are leading to a necessary update of the WHO 2016 classification in the upcoming 5th edition of the WHO Classification of Tumors of the Central Nervous System in 2021 [9,10]. The fast advancements in biological sciences are promoting to the continuous discovery of promising biomarkers and new drug targets, and therefore an acceleration of the revision process is required. Accordingly, cIMPACT-NOW (Consortium to Inform Molecular and Practical Approaches to CNS Tumor Taxonomy) was established in order to provide regular updates [10,11].

## 3. Low-Grade Gliomas

### 3.1. Activated Molecular Pathways in LGGs

In contrast to adult LGGs, the majority of pLGGs feature a unique activated signal transduction pathway, the RAS/MAPK (mitogen-activated protein kinase) pathway. In each case of pLGGs, only one variant genetic event could be observed, uniformly leading to the activation of the RAS/MAPK pathway. Therefore, pLGGs are regarded as “one-pathway diseases” [4]. The RAS/MAPK pathway can be affected by numerous inhibitor agents targeting different levels of the activated pathway. At present, these inhibitor agents are under investigation in different phases of human clinical trials. Whenever possible, it is essential to identify the exact genetic alteration by tissue sample analysis to initiate targeted therapy.

The most common tumor predisposition syndrome is neurofibromatosis type 1 (NF1), which is the consequence of a germline mutation of *NF1*, a tumor-suppressor gene located on chromosome 17. The *NF1* gene normally produces neurofibromin, a negative regulator of RAS protein. Failure of its suppression will activate RAS [12]. It is known that 10–15% of children with NF1 will develop optic pathway LGG and an additional 3–5% will present LGG outside of the optic pathway, the latter with worse prognosis [13,14]. LGG in NF1 could regress spontaneously, but in case of deterioration, as a first-line treatment, chemotherapy is applied (vincristine plus carboplatin or vinblastine in monotherapy) with strict avoidance of irradiation and without pretreatment biopsy [15,16,17]. In NF1, LGGs containing genetic alterations activating the RAS/MAPK or other pathways and HGG could also occur. Recently, CNS tumor biopsy has been highly indicated in NF1, at least for a focused testing of mutations and to choose the best treatment option [17].

The BRAF–KIAA1549 translocation is the most common molecular alteration in pLGG, which is the consequence of focal gains at 7q34, the location of the *BRAF* gene [4,18]. Due to the loss of the N-terminal regulatory domain of BRAF, downstream activation of the RAS/MAPK signaling pathway occurs [19]. BRAF–KIAA1549 is most commonly observed in pilocytic astrocytoma and in tumors arising in the posterior fossa [4]. Overall, 35% of pLGGs harbor this mutation [13]. The common cerebellar localization and a well-circumscribed behavior, especially in pilocytic astrocytoma, make the complete surgical removal amenable and predispose to an excellent outcome without additional treatment. LGGs harboring the BRAF–KIAA1549 translocation in other locations with incomplete surgical removal still have a better outcome compared to those lacking this genetic alteration [4,20]. Despite this, for tumors located in deep areas unfeasible for complete resection, progression could occur, and this may force additional therapy.

The BRAF V600E mutation in which a valine is replaced by glutamic acid at position 600 makes the MAPK/RAS pathway constitutively active. The BRAF V600E mutation as a molecular background for progression most commonly occurs in pleomorphic astrocytoma (77.8%), diffuse astrocytoma (43.5%), and ganglioglioma (49%) and is less common in pilomyxoid astrocytoma (13.3%), pilocytic astrocytoma (3%), and other LGGs, accounting for 15 to 20% of all pLGGs [21,22]. Supratentorial tumors, in contrast to cerebellar lesions, more commonly harbor BRAF V600E than the BRAF–KIAA1549 translocation [4]. pLGGs with the BRAF V600E mutation have a higher rate of local recurrence and a low overall survival rate after conventional treatment (irradiation, chemotherapy), despite their benign phenotype [21]. BRAF V600E-mutated pLGGs with the presence of the CDKN2A (Cyclin-Dependent Kinase Inhibitor 2A) deletion, more commonly described in pleomorphic xanthoastrocytoma, have a highly increased risk to transform to HGG in one or two decades [4,23]. As the co-occurrence of CDKN2A deletion has only recently been described, the real genetic alteration leading to progression is still under debate [24].

Activation of the RAS/MAPK pathway less frequently occurs at the receptor tyrosine kinase (RTK) level. FGFR1 (Fibroblast Growth Factor Receptor 1) plays a key role in signal transduction through the activation of its intramembranous domain [25]. FGFR1 activating alterations are based on three different mechanisms: FGFR1 mutations (p.N546K, p.K656E), FGRFR1–TACC fusion, and FGFR1-TKD (tyrosine kinase domain) duplications [22,26,27]. All these mechanisms could be assigned to typical histological subtypes, but non-exclusively. FGFR1 mutations most frequently (20%) accompany DNTs, other glioneuronal tumors, and tumors in midline brain structures, but they rarely occur in pilocytic astrocytomas and oligodendrogliomas [4,13]. The FGFR1–TACC fusion most commonly occurs in pilocytic astrocytomas with a cystic lesion. FGFR1-TKD duplications, similarly to FGFR1 mutations, are most commonly (2–3%) present in DNTs and other glioneural tumors restricted to a cerebral hemisphere [4,13,26]. FGFR1 alterations commonly cause the upregulation of the PI3K/Akt/mTOR pathway [4]. FGFR1-mutated pLGGs have worse prognosis, but it is unclear if this is a consequence of reduced resection due to their midline localization or of the mutation itself [28]. NTRK (Neurotrophic Tyrosine Receptor Kinase) fusions, activating the same pathways described for FGFR1 activation, less frequently occur in LGG [4]. An ALK (Anaplastic Lymphoma Kinase) gene fusion has also been described in rare cases of gliomas, resulting in the activation of similar pathways. ALK fusion is mostly observed in infantile gliomas [29].

Other genetic alterations are also observed in pLGGs. MYBL1 and c-MYB have less significant effect on survival, and there are typical mutations of adult LGGs identified in older children (IDH1, IDH2, H3F3a), with incompletely identified prognostic significance [30]. Recent cIMPACT-NOW updates suggest creating a “pediatric-type” diffuse glioma group comprising histologically diffuse gliomas with BRAF V600E mutation (without CDKN2A/B homozygous deletion), FGFR1 alteration, or MYB and MYBL1 rearrangement [31].

### 3.2. Diagnostic Aspects

The identification of the central role of the RAS/MAPK and PI3K/Akt/mTOR pathways in pLGGs has important therapeutic implications. Several drugs targeting activated RAS/MAPK or PI3K/Akt/mTOR will rewrite the first-line treatment approach for the involved tumors. The application of targeted treatment for activated RAS/MAPK or PI3K/Akt/mTOR pathway will be enabled by the improved diagnostic methods. Although next-generation sequencing and other high-cost diagnostic methods may provide deep and profound insight into the genetic changes in pLGGs allowing a better understanding of the mechanism of development and progression of these tumor types, there are simple, well-known, widely used methods for the proper identification of therapeutic targets. Immunohistochemistry could be used to detect the BRAF V600E mutation, and FISH (fluorescence in situ hybridization) is an excellent method for the identification of BRAF, FGFR1, ALK, NTRK gene fusion or copy number alterations (and, for prognostic purpose, co-occurring CDKN2A deletion) [4].

### 3.3. Targeted Therapy

The first results of targeted treatments of pLGGs are promising. The first generation of BRAF inhibitors (dabrafenib, vemurafenib), targeting BRAF V600E mutation, were taken over from melanoma treatment. In a retrospective study, collecting the data of 56 pLGG patients worldwide, treated with BRAF inhibitors (dabrafenib or vemurafenib), the objective response rate was 80%. The 2-year PFS (progression-free survival) of the 28 patients treated continuously for more than 10 months was 81.6%, and no further progression was observed in those who received continuous therapy up to 5 years [32]. A prospective phase 2 trial of 32 pLGG patients with BRAF V600E mutation showed 44% overall response rate, 85% one-year progression-free survival, and acceptable toxicity [33]. Interestingly, BRAF inhibitors cause the parallel activation of the RAS/MAPK pathway in LGGs with BRAF–KIAA1549 translocation or wild-type BRAF, which emphasizes the importance of clarifying the exact alteration of the Ras/MAPK pathway before the initiation of therapy [34]. In the signal transduction pathway, MEK activation takes place downstream of BRAF activation. MEK inhibitors could be used in cases of pLGGs that cannot be treated with BRAF inhibitors. Selumetinib is in the most advanced phase of testing, but there are also results in human patients with trametinib. In 25 pediatric patients with BRAF–KIAA1549 fusion or BRAF V600E mutation, 36% of the patients showed partial response, and in 25 NF1 patients, the partial response rate was 40%, and 68% of the patients had no progression in 4 years of median follow-up [35].

The rare presence of NTRK alterations in pLGGs justifies the administration of tyrosine kinase inhibitors such as entrectinib and larotrectinib, for which phase 2 studies are still ongoing (NCT02637687, NCT02650401).

The identification of a common activated signal transduction pathway and encouraging first results obtained by its targeted treatment, accompanied by relatively rare and well-tolerable side effects, are expected to lead to the administration of molecularly targeted treatments for pLGGs as a first-line treatment.

## 4. High-Grade Gliomas

### 4.1. Genetic Alterations in pHGG

The emerging knowledge about the molecular background of different HGGs has partially overwritten the former cell-of-origin-based WHO2007 classification of pHGGs, but in the recent WHO2016 classification, the new information was still not completely included. The WHO2016 classification has already replaced DIPGs from low-grade gliomas to a special group of HGGs, called H3K27M-mutant diffuse midline gliomas, harboring a unique molecular feature (K27M histone mutation), with extremely aggressive behavior, originating from a midline structure (thalamus, brainstem, spine, etc.) [5]. Recent molecular findings have created a highly heterogenous, potential group of hemispheric pHGGs, in contrast to diffuse midline gliomas [36]. There are two potential distinct groups of pHGGs: constitutional mismatch repair deficiency syndrome-based hypermutant glioblastoma multiforme, enabling a special therapeutic approach, and infant high-grade gliomas in children less than 3 years old, with a more favorable outcome [37,38].

The majority of pediatric diffuse midline gliomas harbor mutations at position 27 (K27M) in H3F3A (cca. three-fourth) and in *HIST1H3B*/C genes (cca. one-fourth) coding histone 3 variants (brain stem: >90%, thalamus: cca. 50%, spinal cord: cca. 60%) [39,40]. Methylation of histone H3 on lysine 27 (H3K27M) by the polycomb repressive complex 2 (PRC2) with its catalytic domain, a histone methyltransferase protein, EZH2, is the initiating element for silencing of a genomic region [41]. PRC2 activity is hindered by the K27M-mutant histone 3 protein through sequestration of its catalytic subunit EZH2, leading to a globally decreased H3K27 trimethylation (H3K27me3) [42]. Decreased H3K27me3 results in the activation of the silenced genomic region, responsible for tumorigenesis. H3.3 mutation occurs in all midline gliomas typically affecting children with a median age of 7.4 years and has a very poor outcome (median survival 9.2 months). In contrast, H3.1 mutations almost exclusively occur in DIPGs, affecting children with a median age of 5.1 years, have a better response to irradiation and a slightly better prognosis (median survival 15.0 months), and often co-occur with *ACVR1* mutation [39]. Recently, it has become known that H3 K27M mutations are not exclusive to HGGs but may also occur in rare cases of low-grade midline gliomas and posterior fossa ependymomas with unknown prognostic significance [6]. 

Hemispheric pHGGs comprise another highly heterogenous group, occurring in older age (10 to 25 years) with a slightly better survival compared to other pHGGs. They should be consider as a distinct entity [6]. Another mutation in the *H3F3A* gene at position 34 (G34V/R) in one-third of hemispheric pHGG cases, a high percentage of *TP53* mutations (>85%), *ATRX* loss (95%), and *MGMT* (O6-methylguanine-DNA-methytransferase) promoter methylation (75%) are typical, almost exclusive features of this localization [36]. This distinction does not appear in the latest WHO2016 classification yet.

A small molecular subgroup of pHGGs occurring in older adolescents, which carries the features of adult HGGs (IDH1/2 mutations), could be regarded as the younger age spectrum of adult HGGs [43]. The residual heterogenic group of pHGGs (H3/IDH wild-type) compose more subgroups, e.g., with *MYCN* amplification, with mutation or amplification in tyrosine kinase receptor genes (PDGFR, EGFR), with fusions involving MET or NTRK1–3 genes, and with BRAF V600E mutations. The latter, histologically resembling pleiomorphic xanthoastrocytoma, compose 5 to10% of pHGGs and are associated with a slightly better survival compared to other pHGGs [6,44].

Genetic predisposition of constitutional mismatch repair deficiency syndrome (CMMRD) results in a small subset of pHGGs with a high mutational burden. These pHGGs have a unique sensitivity to immune checkpoint inhibitors (detailed later in the manuscript) by presenting a high load of T cell-activating antigens [37].

A special group of pHGGs called infant high-grade glioma has a more favorable outcome [38]. At present, they are classified in three separated groups based on their molecular features: hemispheric RTK-driven tumors, hemispheric Ras/MAPK-driven tumors, and midline Ras/MAPK-driven tumors. The best survival rate is found for hemispheric Ras/MAPK-driven tumors (10-year OS is 93.3% after minimal therapy beyond surgery,) followed by hemispheric RTK-driven tumors. Their 5-year OS is different depending on the accompanying driver mutations, i.e., ALK, NTRK, ROS1, leading to 5-year OS of 53.8%, 42.9%, and 25.0%, respectively [29].

The current common therapeutic approach to pHGGs is maximal surgical resection, whenever feasible with irradiation, especially above the age of 3 years. Additional chemotherapy (temozolomide, CCNU, etc.) with or without bevacizumab is also administered (especially for hemispheric HGGs, similarly to adult cases). However, its therapeutic impact beyond irradiation is highly questionable [45]. Treatment of pHGGs, except for some small special entities, could be regarded as highly ineffective.

### 4.2. Immunotherapy

Novel therapeutic approaches should be considered. As HGGs are generally highly immunogenic tumors, immunotherapy could be the next powerful therapeutic approach for highly chemo- and radioresistant HGGs. The four main components of this therapeutic modality are immune checkpoint blockade, chimeric antigen receptor (CAR)-T therapy, vaccine therapy, and immunovirotherapy [42,46].

Immune checkpoint inhibitors play a role restoring CD8+ T cell activation, which is decreased in the tumor microenvironment. Immune checkpoint inhibitors target T cell response modulator or inhibitor co-stimulation molecules, such as cytotoxic T lymphocyte-associated protein 4 (CTLA-4) and antibodies blocking checkpoint programmed cell death (PD-1) or its ligand (PD-L1) [47]. There are hypermutant GBM cases with germline CMMRD, where a radiologically and clinically proven response occurred after anti PD-1 inhibitor (nivolumab) treatment [37]. Unfortunately, the lack of a high tumor mutational load or a low level of PD-L1 expression make checkpoint inhibition ineffective as a monotherapy for different tumor types [48].

CAR-T cell therapy applies special T cells originating from the patient. These T cells have hybrid receptors with a double function: they bind specific antigen proteins of the tumor cells and activate other T cells toward a special tumor antigen [49]. In diffuse midline gliomas, both B7-H3 and GD2 (disialoganglioside) are highly expressed [50,51]. B7-H3 is a type of immune checkpoint molecule mainly expressed by T and B cells [52]. B7-H3 participates in T cell-mediated adaptive immunity either in a costimulatory or in a coinhibitory way [53]. Expression of B7-H3 in pediatric CNS tumors correlates with tumor grade. High B7-H3 mRNA expression is associated with more unfavorable survival in several tumor types [54]. Accordingly, DIPGs have high B7-H3 mRNA expression [50]. At present, a phase 1 study is ongoing with CAR-T cells to target B7-H3, given intracavitary directly into the CNS [55]. The well-known target of neuroblastoma, GD2, is also highly expressed in DIPGs, which makes it a potential therapeutic target [51]. because of the presence of the BBB (blood–brain barrier), antibodies are unable to reach their target lesion in the CNS after systemic administration [56]. However, in orthotopic xenograft mouse models of H3K27M-mutant patient-derived diffuse midline gliomas, a significant therapeutic effect of CAR-T cells targeting GD2 was shown after systemic administration [51]. CAR-T cell therapy provides a great hope; however, its price and the time-consuming T cell production are significant hindrances for its application. CAR-T cell therapy could have serious side effects, derived from systemic inflammatory immune activation by the elevation of proinflammatory cytokines (especially, IL-6, IL-10, IFN-gamma), called cytokine release syndrome (CRS) [55]. Neurotoxicity, a possible lethal complication of CAR-T cell therapy, has been recently described with a special term. i.e., ICANS (immune effector cell-associated neurotoxicity syndrome). In contrast to intravenous administration, the direct administration of CAR-T cells to the CNS may have a decreased toxicity [55].

The third possible method of immunotherapy is vaccination therapy. Cancer vaccines comprise several types: cellular (immune, tumor), peptide or protein, and genetic vaccines. Effective vaccines targeting tumors have to overcome the evolved tolerance of immune system against tumor cells. The two main steps in this process consist in providing a suitable amount of antigen to dendritic cells and in allowing postvaccination expansion [47]. There is a contrast between successful preclinical studies using vaccines directed against CNS tumors and their frustrating inefficiency in early clinical trials. At present, the most promising approaches after preclinical studies are phase 1 studies targeting the H3K27M mutation in diffuse midline gliomas [57,58].

Another promising form of anticancer immunotherapy is immunovirotherapy. This directly targets the tumor lesion by the local application of a genetically engineered oncolytic virus that promotes an antitumor immune response beyond direct infection and lysis of tumor cells. The first successful phase 1 study was finished recently in 11 recurrent/progressive supratentorial pHGG patients. Replication-deficient neurotrop genetically engineered oncolytic herpes simplex virus type 1 (HSV-1) G207 was administered intratumorally through multiple intratumoral catheters, in different amounts of plaque-forming units (PFU), with or without a single dose of focal irradiation (5Gy). The median survival was 12.2 months compared 5.6 months of historical controls, and four patients survived for more than 18 months. In the absence of a previous HSV1 infection, a dramatic increase in tumor-infiltrating lymphocytes was observed after seroconversion [46].

### 4.3. Circumvention of the Blood–Brain Barrier

The BBB is one of the most significant hindrances to effective treatment. Several approaches exist to disrupt the BBB. There are drug-based (mannitol, RMP-7, regadeneson), techniques, such as magnetic resonance-guided laser ablation (MR-gLA), Cranial Implantable Ultrasound, MR-guided focused ultrasound (MRgFUS). All of them are characterized by promising outcome in early-phase trials, but inefficiency in advanced phases [59].

Another promising approach to overcome the blood–brain barrier is the direct administration of anticancer treatments to the brain parenchyma at the tumor site by catheter injection, thereby generating a high local drug concentration with limited systemic exposure [60]. Pressure gradient, induced by interstitial infusion, generates fluid convection within the brain parenchyma, which amplifies the distribution of high-molecular-weight proteins and other large or small molecules [60]. This phenomenon, called convection-enhanced delivery (CED), is used for spreading the drug. By an indwelling catheter, CED was used in DIPGs for the local administration of radioimmunotherapy (radiolabeled antibody ^124^I-8H9 targeting the B7-H3 antigen) and chemotherapy (carboplatin) combined with a histone deacetylase inhibitor (sodium valproate). Implementation of the so-called Renishaw Drug Delivery System (RDDS) enables multiple administrations over months or years in awake patients [61]. The maximum length of 10 h enables one to infuse up to 10 mL of fluid, resulting in a distribution volume of cca. 30 mL. This kind of approach has increased the overall survival by more than 6 months, with safe administration, and in the long term, has resulted in an excellent quality of life [61].

## 5. Conclusions

The recent advances in understanding the molecular background of pediatric gliomas are facilitating the application of effective drugs targeting the Ras/MAPK pathway, with fewer side effects in the case of pediatric low-grade gliomas, thus substituting harmful chemotherapy and irradiation. In the case of pediatric high-grade gliomas, understanding the molecular mechanism sustaining tumor progression and implementing recent advances to overcome the BBB may open novel therapeutic windows to treat these devastating diseases. The most promising approach is based on the highly immunogenic features of high-grade gliomas, which may be utilized by the reactivation of the ineffective self-protecting immune mechanisms or by applying other mechanisms such as CAR-T cell therapy or vaccination.

## Data Availability

Not applicable.
